# Unblocking High-Value Botanical Value Chains: Is There a Role for Blockchain Systems?

**DOI:** 10.3389/fphar.2019.00396

**Published:** 2019-04-24

**Authors:** Michael Heinrich, Francesca Scotti, Anthony Booker, Martin Fitzgerald, Ka Yui Kum, Katja Löbel

**Affiliations:** ^1^Research Group Pharmacognosy and Phytotherapy, UCL School of Pharmacy, London, United Kingdom; ^2^School of Life Sciences, College of Liberal Arts and Sciences, University of Westminster, London, United Kingdom

**Keywords:** blockchain, herbal medicines, traditional medicines, sustainability, value chains, quality (herbal medicines)

## Abstract

Blockchain systems are a fast emerging and a currently widely discussed novel strategy for a decentralized cryptographically enhanced digital ledger recording transactions among stakeholders. This perspective paper looks at its potential uses in the context of high value and mostly low volume botanical material traded globally and used as medicines, health foods, in cosmetics and other applications. We offer a perspective on key areas in the supply of such products globally and how blockchain systems may help in sustainable sourcing, quality assurance, and in tackling supply problems in cases of complex multiherbal preparations. Both open and closed blockchain systems are feasible, and it seems likely that, at least in the initial development, closed ones are the main ones to be utilized. While blockchain’s potential is not yet clear, the examples presented here highlight the opportunities of this new technology.

## Introduction

**FIGURE 1:**
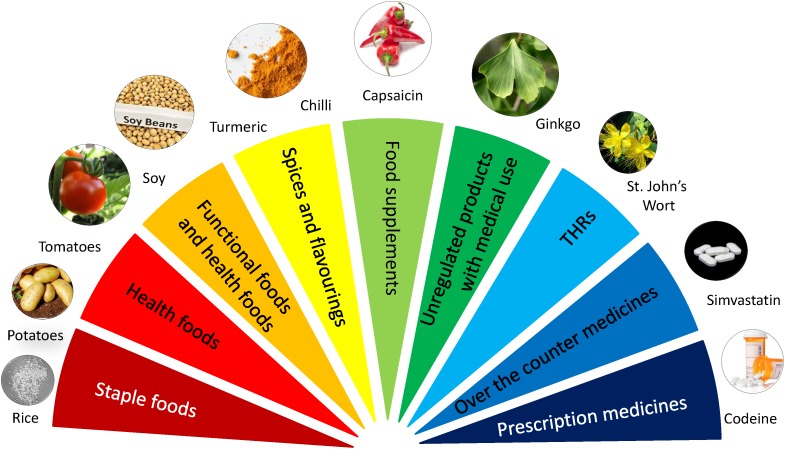
The food-medicine interface: where to draw the line?

Concerns about the quality and authenticity of botanical material used in medicines and food have existed at the very least since the start of international trade in such materials. This includes materials that are used as high value foods, food supplements, cosmetics or herbal medical products. As highlighted in Figure 1, a broad array of products exist which rely on the supply of primary material through complex value chains.

The global trade in high value botanical products has increased enormously bringing the challenges associated with the supply of such products to the center of the debate in ethnopharmacology and medicinal plant research. Very often the focus has been either on addressing specific quality problems (e.g., adulteration and poor production systems) or on solving specific concerns in social or economic terms. These challenges were generally addressed by defining quality standards, for example, in a pharmacopeia (for medical products) or in industrial QC standards (esp. for food and cosmetic products). These supply systems also gave rise to a number of specific concerns including equitable benefit-sharing/responsible sourcing and sustainable supply, especially as it relates to protected or threatened environments. In many cases this had been seen as a supply issue, but at least since 2012 an increased awareness has developed on the wider role of such herbal materials’ value chains in a globalized market (Booker et al., 2012).

This links to a series of core characteristics of this sector, which include:

– The material may be collected from the wild or cultivated and the supply may be based on a mix of the two
– Presence of a large number of primary producers, who often produce small quantities of *materia prima*
– These materials enter complex value chains, often requiring middlemen to link to international purveyors
– Knowledge about the product characteristics along these value chains is often limited
– Lack of knowledge about and often poor practice relating to endangered/overharvested species
– Dramatic increase in price from primary producer to end users
– The regulatory status and subsequent commercial expectations differ in various markets. A specific botanical drug may yield a licensed medicine in one market and an unregulated food supplement in another one

While Booker et al. (2012) highlighted the opportunities arising from analyzing value chains using a transdisciplinary approach, more recent developments may offer a new solution – blockchain systems, a technology now widely seen as an opportunity to solve the challenges arising from the fast changing socio-economic framework of how societies exchange information and goods (trade). This is exemplified by the launch of *Frontiers in Blockchain* in September 2018, which publishes rigorously peer-reviewed research covering theory and applications of blockchain and blockchain-related technologies^1^. Therefore, in this perspective article we explore the opportunities in using blockchain systems in a sector that is dominated by many stakeholders, and specifically, how this can help to ascertain equitable benefits to producers, sustainable sourcing and production, and the best quality of the final products.

## The Concept of Blockchain

Key in the exchange of goods and information is knowledge along a value chain of what is being exchanged including specific requirements relating to a products’ composition, quality, source, and many other relevant data, traditionally stored in analog ‘ledgers.’ Blockchain (BC) – as opposed to the “traditional” centralized system – is a system of *decentralized data storage* that is built on a net of users, each of which can become a node of the network (Figorilli et al., 2018). Any new information (stored in blocks) is linked to other blocks containing previous data, forming a chain of information and thus a history of interactions which is stored and copied in all the nodes within the network. Validation prior to incorporation into the BC is a key feature. Therefore, each computer is an official custodian of one copy of that blockchain. There will be as many identical copies of the blockchain as there are computers in the network, allowing for a more efficient identification of corrupted data and their control. Data on the BC is unmodifiable as, in practice, all that can be modified is a single copy of the BC, which then, compared to all the other copies, will be different, and could be easily singled out and flagged as wrong/corrupted. There seems to be no shortcut to modify more than one copy at one time (Carson et al., 2018; Saberi et al., 2019).

On the other hand, it is easy enough to grasp how a “traditional” centralized system suffers data corruption. A failure of the main computer, or corruption of data stored on it, results in a spread of the error/modification to all the receivers connected to Central. Therefore, data security depends on the security of the central system. This of course translates into fear and therefore considerable investment in security systems. Blockchain subsequently becomes a revolutionary way to shift the issue of data security from external “security providers” to an internal network of “trust,” which is free of charge.

In 2018 Galvez et al. (2016) argued convincingly, that there are enormous opportunities (but also major obstacles) for using blockchain approaches in the food sector. Their focus is in essence on the optimisation of industrial processes and on the potential of blockchain system from the perspective of the large industry. Early stages of production were not covered in any detail. Obviously, they also did not cover the additional challenge of securing that the end products will have a therapeutic benefit. An herbal medical product will not only have to comply with label claims, but it also must have a composition and quality assuring therapeutic benefits.

**FIGURE 2:**
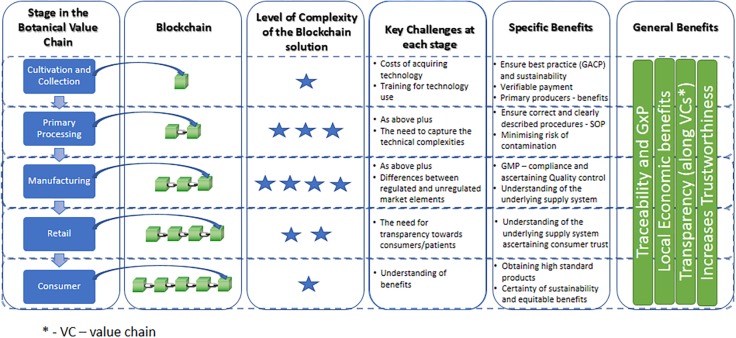
A general overview of the various stages of value chains of botanical products and how these could be managed using blockchain systems. Levels of complexity range from low (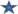) to very high (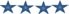).

This perspective article introduces how BC systems in food/herbal markets will greatly benefit a sector relying on complex transaction networks, often lacking trust in its value chains (Figure 2). The “trust” is based on transparent exchange of unmodifiable data, uploaded and securely stored on the blockchain, bypassing intermediaries in their role of “trust guarantors” between untrusting parties. Blockchain technology provides a safe/trusted platform for interactions between such parties without the need for individual trust to be built. This can be reinforced by the use of smart contracts (SCs) which can be embedded into the BC framework. The use of SCs is a way to efficiently and securely validate a contract in the digital world creating a negotiation, agreed by all involved parties, whose terms are defined and need to be fulfilled to “release” the agreed actions (Carson et al., 2018). In other words, when all the conditions required in the contract are met, the payment to the respective parties will be made automatically, assuring a fair and fraud-free transaction. The benefits of using this technology are:

• Increase in reliability even if the parties do not trust each other
• Increase of economic benefits, eliminating third party involvement for mediation, decreasing costs
• All records saved in the blockchain are available to see and can serve to promote the good practices of the parts
• On the small producer’s side, this technology can help them to receive almost immediate benefits from the commercialization

In the following, we used five areas of major concern as case studies for how such problems might be overcome.

## Case Study 1: Quality Considerations in Herbal Value Chains

The quality assurance of herbal medicinal products is a more complex process than assuring the quality of pharmaceutical products. Natural products are typically highly variable in their chemical composition and any quality assessment must take into account the long, diverse and often obscure value chains from which these products originate. While such quality aspects are commonly controlled in products holding a full pharmaceutical manufacturing license and the quality requirements are well-established using pharmacopoeial methods, quality assurance cannot be guaranteed, for poorly or unregulated products. Food standards also exist.

The quality and safety of a product in a short chain, i.e., the plant is grown and used as a medicine in close proximity or even grown and used by the same person, is relatively simple to effectively control, assess, and manage. However, the same plant when destined for use as a medicinal product or food supplement on the national market or as an export product presents far greater risks and consequently greater challenges to overcome, requiring the collection of complex sets of data (Booker et al., 2012). Authenticity, purity, and compliance with the allowed maximum levels of contaminants (microbiological, heavy metals, and toxic substances) are generally controlled at a very late stage and importantly, only a small share of such botanical products is produced under rigorous pharmaceutical quality control.

### Heavy Metal Contamination

Herbal products are prone to heavy metal contamination for two main reasons: pollution and natural accumulation. Pollution caused by heavy industry is a particular problem in fast developing countries, e.g., China and India. Although this situation has begun to be addressed in some countries, the legacy of years of pollution finding its way into agricultural land via the rivers and waterways will take a long time to remediate. Very often little is known about pollution levels in specific areas. Secondly, some plants naturally accumulate heavy metals, e.g., cadmium in *Perillia* sp. or in *Hypericum perforatum* L., and in some areas heavy metals are found in the subsoil, e.g., cinnabar in rock formations leading to high levels of mercury in certain species. Only through careful selection of cultivation sites or strict controls on wild collection can these problems be minimized.

There is little need for pesticides in the short chain, but global supply and consumer expectations often require intensively farmed crops that need higher levels of pesticides to produce high yields of the desired product commonly at a low cost. In addition, many of these chemicals may be prohibited under European legislation. Only through careful management of the value chain and detailed training as to which pesticides may be used and at what quantities can we hope to keep pesticides’ residues within allowable limits.

### Mycotoxin

Aflatoxins (mycotoxins produced by *Aspergillus* spp.) are highly toxic and carcinogenic, but are regularly found in botanical materials esp. in the food industry (Liu and Wu, 2010). Certain types of products are more at risk than others and analysis has shown high levels of aflatoxins, for example, in wheat, barley, maize, and nuts, but little has reached the general media regarding the problems encountered in herbal medicine manufacture (Williams et al., 2004; Azziz-Baumgartner et al., 2005; Liu and Wu, 2010). This is partly due to a lack of robust regulation in many importing countries but also due to a lack of awareness within the value chain about these problems and their lethal potential (Williams et al., 2004).

Transportation, storage, or processing are key areas where blockchain management may help to minimize risks. Development of aflatoxins in a short value chain is unlikely, problems arise when fungal molds are given the right conditions to evolve, typical of global complex value chains. Procedures employed at a local level, utilizing small quantities of materials, may be entirely unsuitable for the global distribution of large consignments and it is vital that this is recognized and the correct processes and procedures are developed, implemented, and controlled.

### Unintentional Adulteration: Pyrrolizidine Alkaloids in Saint John’s Wort (SJW) Products (*H. perforatum* L.)

In February 2016, six batches of SJW THR products sold in the United Kingdom, produced in 2013, were recalled due to having levels of pyrrolizidine alkaloids (PAs) above-the-European Medicines Agency’s recommendations. These compounds are associated with potentially very serious health risks (liver toxicity and cancer) (Letsyo et al., 2017). PAs are not produced by *H. perforatum*, but are found in certain species especially Boraginaceae and some Asteraceae. Therefore, their presence in SJW herbal preparations is likely due to the presence of high PA yielding weeds (such as *Senecio*, *Crotalaria*, *Echium* spp., and others), which had been growing in the SJW fields and unintentionally harvested along with the desired herb. It is currently difficult for authorities to identify sources of the adulterated material and where the incriminated batches ended. A blockchain system, recording all the movements of a good, from seed purchase to packaging facilities, retailers, and perhaps, final customer purchase, would provide easily accessible information to restrict the recall warning to a targeted area (e.g., South West United Kingdom), leading to a faster and more efficient removal of those products from the market/households. In addition, authorities and general audience would have the possibility to flag the incriminated batches on the blockchain itself.

### Intentional Adulteration: The Case of Food Dyes in SJW Products

Studies on the quality of SJW products on the market (Frommenwiler et al., 2016; Booker et al., 2018), found that some samples contained undeclared food dyes, which are likely to have been introduced with the purpose of mimicking hypericin absorbance in samples containing a considerably lower concentration of hypericins than good quality ones (Booker et al., 2018).

The above examples are by no means a definitive list of quality concerns in herbal medicines, but they present the majority of risks that can be attributable to lack of knowledge and good practice along poorly managed value chains. BC systems are ideal to monitor the individual steps from the primary production the initial processing and then the production of a finished product. BC systems offer traceability allowing for a faster identification of the source (s) of a problem, e.g., identification of where along the chain quality control measures were lacking. Data on good agricultural and collection practices (GACP), good manufacturing practice (GMP) can and need to be included in this. Although we have the capabilities to detect a wide array of quality failures using the latest analytical techniques, BC systems will facilitate the traceability of quality parameters, with the initial steps requiring far lower level of validation, than the subsequent ones.

## Case Study 2: Sustainable Sourcing

At present there are many cases of sustainability problems (Schippmann et al., 2002), which resulted in withdrawal of some products from regulated market (e.g., *Drosera* spp. previously used in European phytomedicine) or again in adulterations.

In the 1970s African cherry, *Prunus africana* (Hook.f.) Kalkman, became popular as a phytomedicine for its positive effect in benign prostatic hyperplasia (BPH) (Stewart, 2003; Bodeker et al., 2014). Since then the approval of a patent and several licenses, exponentially increased the demand leading to vast illegal harvesting. High rates of poverty in countries supplying *P. africana*, a crop playing an important role in the subsistence of villagers, together with the lack of regulation of the governments involved in the value chains (both on the side of the primary producer and the end consumer) drove the overexploitation of *P. africana* out of control. This resulted in it becoming an endangered species (Bodeker et al., 2014).

*Warburgia salutaris’* case (*W. salutaris* (G. Bertol.) Chiov.) is more dramatic, as this medicinal plant has not only been classified as Endangered in Swaziland (Dludlu et al., 2017), but its population is steadily shrinking due to illegal trading and uncontrollable harvesting. Its increasing popularity as a traditional medicine contributed to its overexploitation, resulting in its classification as Extinct in the Wild in Zimbabwe (Botha et al., 2004; Dludlu et al., 2017).

Based on recent changes in the supply of goods, increasing awareness and the many concerns associated to- the dramatic environmental changes, consumers are demanding more information about the products they are purchasing. According to the Ethical Consumer Markets Report 2017 the main growth in consumers’ spending was driven by sustainability certification of the goods, as Certified Sustainable Seafood MSC and Rainforest Alliance (Ethical Consumer Research Association [ECRA], 2017). The use of BC technology can firstly aid in monitoring collection and processing of these materials, providing important baseline data on what is actually harvested/collected. This can improve sustainable production, as all the detailed information of the product will be available for the participants of the value chain. If sustainable production systems are developed, BC systems can certify the sourcing in these production systems. Geotracking and information on the initial processing can prevent overexploitation and help defining better production systems.

## Case Study 3: Benefits to Producers of the *Materia Prima*

The benefits to primary producers remain a hotly contested area. Very often financial benefits are limited. Whereas relatively well established farming businesses in the developing South may be able to connect to international trade, small farmers and farm workers are unlikely to have resources or capabilities to form business relationships with manufacturers and retailers of herbal medicinal products in the industrial North. However, for farmers and primary producers who established more sophisticated operations and managed to form these relationships with international partners, there are advantages for producers, manufacturers, and end-consumers of these products.

As Booker et al. (2012) highlighted for turmeric, in a traditional value chain, whereby the farmer sells the product at an auction house, the farmer has relative autonomy over how the crop is cultivated and could sell it for the highest price available. However, turmeric price varies considerably from year to year and if the price is low the farmers often choose to store the crop rather than sell, resulting in long-term storage under poor conditions, using chemical treatments (pesticides and fungicides) to prevent deterioration with serious implications for the quality of the end product.

In the second model presented, a company established a direct contract with the farmer, agreeing on a price for a quantity of raw material early on in the process and, once cultivated, transferred directly to the product manufacturer. This system demonstrated some benefits to the farmer as it provided better market stability, knowledge of the quantities to grow and an agreed price which could be negotiated beyond the typical market price. In return, the manufacturer could exert greater control over the chain and request specific private standards (organic production, national standards) (Booker et al., 2012). Moreover, any welfare benefits passed to the farmer in terms of employment stability, price stability or price premiums could be utilized for companies’ overall marketing and advertising strategy. The potential benefits to the end consumer were access to higher quality and more socially responsible products.

Currently there are few checks to ensure that contracts, processes, and procedures are adhered to by either party. Whereas some companies are well established in the market, having built up trusting relationships with partners over a period of years, there is no barrier to prevent a “me too” type marketing strategy but one that lacks the necessary skills and infrastructure to actually produce a product of quality and with little scope for any meaningful welfare benefits.

Here, BC systems will offer transparency both ways; it will be apparent to both consumers and producers what the economic benefits and the resulting values are. It may enable more direct links between (groups of) producers and the relevant companies producing the finished product, either for the national market of origin or the non-domestic destination.

In a very similar way, BC systems can be used to provide evidence for organic and sustainable sourcing. Consumers would acquire a final product knowing exactly where it came from locally sourced, what type of certification it has (reliability) or its production’s social and environmental impact. As BC-stored information is immutable, consumers would purchase products with more confidence as there will be no information bias based on the retailers’ own benefit.

## Case Study 4: Chinese Medicine Market: an Example of Added Complexity Within the Value Chain

Traditional medicinal products as used in traditions like Indian, Chinese, African or American medical systems pose many problems that are similar to those of other value chains of high value botanical products. However, the very different approaches to the use of these resources, based on their unique underlying philosophies, result in some specific challenges, when translated into the globalized and interconnected markets. Crossovers between value chains based on traditional practices to ones that result in globally recognized products are likely. The source material itself (i.e., the species and botanical drug) is often complex, encompassing species or drugs that outside of the country of origin may not be permitted or might be regulated differently, incurring in numerous problems regarding the supply of the correct material. In addition, complex processing of the botanical material in the early steps of the value chain, is often required as it is seen as improving the ingredient’s therapeutic usefulness.

Here, we use Traditional Chinese medicine (TCM) to highlight some key challenges. Over 90% of the TCM herbal market is constituted by formula products, remedies typically containing between three and ten ingredients, often produced in ways which are unique to TCM (Scheid et al., 2009). Consequently, a range of complex development, manufacturing, and retail challenges exist. This dramatically increases potential risks of poor quality, adulteration or other quality problems. Therefore, it is essential for the future of Chinese herbal formulas that, for example, each raw material used is traceable including a documented record of its progressing along the chain. A blockchain would allow to record and verify that the correct source material had been used within the formula and that any primary processing has been carried out according to defined procedures, ideally well-defined industrial standards (i.e., SOPs – standard operating procedures).

Traditional Chinese formula products have had difficulty in finding a stable and secure regulatory position within markets outside of China. This situation arises chiefly because these products are manufactured in Asia to Asian standards, often for Asian markets. When they become global products, the legal requirements of the importing countries are often not or only partially considered. Sometimes commercialization abroad is attempted by straddling the hazy interface between food and medicine.

The TCM formulation “Eight-Gem decoction” comprises eight Chinese herbs, in China recognized as both medicines and food. All the ingredients are commonly used in TCM and widely cultivated. However, the sources of “Eight-Gem decoction” materials are variable. In this specific formulation, some of the materials require extra processing (as described in Table 1). Steaming and frying are not uncommon requirements in TCM preparations, as such steps are meant to change or enhance/limit the activity of that ingredient (Wu et al., 2018).

**Table 1:** “Eight-Gem decoction” – Botanical drugs and their processing.

Drug name (International)	Species	Common name	Optional processing
Ginseng Radix et Rhizoma	*Panax ginseng* C.A. Mey	Renshen/Ginseng	Steaming
Atractylodis Macrocephalae Rhizoma	*Atractylodes macrocephala* Koidz	Bai zhu	Frying
Poria	*Poria cocos* (Schw.) Wolf	Fuling	
Angelicae Sinensis Radix	*Angelica sinensis* (Oliv.) Diel	Danggui/Chinese Angelica	Frying with Chinese yellow wine
Chuanxiong Rhizoma	*Ligusticum chuanxiong* Hort	Chuanxiong	–
Paeoniae Radix Alba	*Paeonia lactiflora* Pall	Bai shao	Frying
Rehmanniae Radix Praeparata	*Rehmannia glutinosa* Libosch	Shu Di Huang	Steaming with Chinese yellow wine
Glycyrrhizae Radix et Rhizoma Praeparata cum Melle	*Glycyrrhiza uralensis* Fisch./*G. inflata* Bat./*G. glabra* L.	Zhi Gan Cao/Fried Chinese liquorice	Frying with honey and Chinese yellow wine

An example of the problem of conversion for different markets is *Rhodiola* spp., a widely used remedy. *R. rosea* L. is the species traditionally used for herbal medicine in Europe while in TCM *R. crenulata* Hook.f. and Thomson is the only accepted species. *R. rosea* products were found to contain *R. crenulata* indicating confusion in species authentication (Booker et al., 2016). Another example is Glycyrrhizae Radix, one of the most frequently used TCM materials (Gancao). Based on the Chinese (2015) and British (2018) Pharmacopeia the current official source species are *Glycyrrhiza uralensis* Fisch., *G. inflata* Bat., and *G. glabra* L. but the United States and Japanese pharmacopeia only accept the first two species (Wu et al., 2015). Other *G.* species and varieties are locally called as “Gancao” but they are not listed in any pharmacopeia.

Using blockchain systems, a complex process which may result in products used in a more traditional context (i.e., within TCM in China) or for a globalized market could be clearly documented providing transparency for suppliers, processors, and end-users.

## Case Study 5: Intellectual Property Questions

Intellectual Property Rights have become much contested in the area of herbal (medical) products and the abidance to international agreements and national laws is now an essential element of any rational development of a new herbal product. As seen throughout history, commercialization of genetic resources and local knowledge without mutually agreed benefit-sharing has led to exploitation and often has stained relationships between countries. Despite the existence of the Convention on Biological Diversity (CBD), Intellectual Property on biodiversity and genetic resources is seldom respected, as the convention does not result necessarily in prosecution. One very recent example is that of maca, *Lepidium meyenii* Walp., a Brassicaceae native of Peru. Its hypocotyl has been exported to China without well-defined and mutually agreed terms and conditions for its development and it is now widely cultivated especially in China for commercial purposes (Heinrich and Hesketh, 2018). China has become the largest maca producer and is now the prime competitor of Peru for maca’s sales. This “transaction” was never released under an agreement on benefit-sharing with the Peruvians (the owners of the natural resource) and it is therefore to be considered illegal, but what can be done now? Boycott Chinese products? That would only be possible if we were to know exactly where each product came from, as food supplements product do not have to state the material provenance. A blockchain could provide the information necessary and make data about each step transparent for authorities in CBD-abiding countries to block the sale of illegal products and for consumers to make informed ethical purchases.

## Conclusion: Blockchain Systems and the Global Trade in Botanical High Value Materials

Food value chains share many challenges and bottlenecks with herbal medicine value chains, and the United Kingdom’s Food Standard Agency (FSA) is already looking into how to implement the food value chains using blockchain technology (for example, in slaughter houses) (FSA, 2018). Additionally blockchain systems in farming and agriculture are being investigated and it is plain to see how the benefits would also apply to herbal production. The question to address is then: “What makes the herbal supply blockchain different from any other cultivated food (coming from abroad)?” and “how do we address these specific issues in the blockchain?”

There are diverse types of BC systems that could be used and, while not exhaustive here we only highlight some general requirements. A closed blockchain technology, with its easily accessible upload and storage of information, could be a feasible answer to address most of the quality issues along the herbal value chain. For example, the raw material can be traced in its provenance and its movement followed ascertaining traceability (e.g., indicating material cultivated in an area with heavy soil pollution). This material would then be subjected by the local (end-market) authority to specific and relevant tests (to make sure it meets the standards of the country where it is being marketed), or otherwise rejected at an early stage. An open blockchain system would impact more strongly on the way how such products are traded reducing the need for intermediaries and middlemen and making transactions very transparent.

One must, however, ask very carefully whether the new drive to use Blockchain systems is just another hype which will not fundamentally change the challenges, or whether it will actually lead to a more sustainable and equitable supply of high quality botanicals. It is not yet possible to assess this at this stage, nor is it possible to decide conclusively on open vs. closed blockchain systems. This paper is a call for exploring opportunities and limitations. Importantly, we will only see a change if this is developed into a sector wide standard. It will not prevent quality problems in general or adulterations, but it will, hopefully, result in a much better traceability allowing all stakeholders to identify and then to resolve problems.

In conclusion, the use of blockchain is now discussed widely, both in areas which are highly regulated by the state (e.g., medicines or specific traditional financial services) and also in less or self-regulated systems (e.g., bitcoinTrade). The trade in high value botanical products represents a prime opportunity to explore the potential and limitations of this technology due to ease of use (if appropriate tools are developed), its versatility and low cost. It will not solve all the problems along the value chains of such products, but offers a mechanism for high quality producers to ascertain their efforts in securing such quality can be documented. Importantly, a Blockchain will not prevent problems directly, but the increased traceability will make it easier to resolve problems. Also, it must be assessed how erroneous entries into a BC will impact on the system’s usefulness.

In general, blockchain systems will provide a much easier way to trace a product from ‘field to fork’ (Figure 2). A certification of compliance with blockchain requirements would offer consumers a range of trusted products. Overall, the numerous challenges along these value chains will become considerably more transparent and, as such, clear guide to what represents best practice in the supply of such high value botanicals. With this perspective paper we hope to open a discussion within ethnopharmacology and natural product research on blockchains’ potential, uses and limitations.

## Author Contributions

MH conceived the original idea for this perspective paper and drafted an outline. FS led the writing of the individual case studies and coordinated much of the writing of the manuscript. AB, MF, KK, and KL contributed case studies and other materials. All authors read and approved the final version of the manuscript.

## Conflict of Interest Statement

The authors declare that the research was conducted in the absence of any commercial or financial relationships that could be construed as a potential conflict of interest.

## Footnotes

1 https://www.frontiersin.org/journals/blockchain#
